# The Integration of Social and Health Sectors in Scotland: An Analysis from the Prism of Different Public Policy Models

**DOI:** 10.3390/jmahp13010008

**Published:** 2025-02-27

**Authors:** Ricardo Correia de Matos, Generosa do Nascimento, Adalberto Campos Fernandes, Cristiano Matos

**Affiliations:** 1 Department of Psychiatry and Mental Health, Centro Hospitalar do Baixo Vouga EPE, 3810-164 Aveiro, Portugal; 2 Ordem dos Enfermeiros, 1700-031 Lisboa, Portugal; 3 Department of Human Resources and Organizational Behavior (IBS), ISCTE—University Institute of Lisbon, 1649-026 Lisboa, Portugal; generosa.nascimento@iscte-iul.pt; 4 National School of Public Health (ENSP), Universidade Nova de Lisboa, 1600-560 Lisboa, Portugal; adalberto.fernandes@ensp.unl.pt; 5 Atlântica School of Health, Atlantic University, 2730-036 Barcarena, Portugal; cristianom@uatla.pt; 6 Instituto de Saúde Ambiental (ISAMB), Faculdade de Medicina, Universidade de Lisboa, 1649-028 Lisboa, Portugal; 7 Laboratório Associado TERRA, Faculdade de Medicina, Universidade de Lisboa, 1649-028 Lisboa, Portugal; 8 Nursing Research, Innovation and Development Centre of Lisbon (CIDNUR), Nursing School of Lisbon (ESEL), 1600-096 Lisboa, Portugal

**Keywords:** integrated public policy, integrated care, healthcare sector, social sector

## Abstract

The integration of health and social care has been a key focus in Scotland, driven by demographic changes, rising healthcare costs, and the need for more efficient service delivery. The Public Bodies (Joint Working) (Scotland) Act 2014 sought to formalise this integration by restructuring governance and service provision to improve coordination between health and social care sectors. Despite these efforts, challenges remain in fully achieving the intended outcomes of the integration. This study analysed Scotland’s integrated health and social care through the theoretical frameworks of public choice, institutionalism, and functionalism. The objective was to examine policy drivers, structural mechanisms, and governance implications, providing insights into the broader impact of integrated care reforms. A qualitative research approach was employed, synthesising data from peer-reviewed literature, government publications, and policy documents. The findings on integration were systematically examined through the lens of each public policy model, allowing for a nuanced analysis of how Scotland’s approach to integration aligns with and diverges from these frameworks. A literature search was performed on PUBMED, Google Scholar, and Scottish government portals. While integration improved coordination and service delivery in some areas, limitations in funding allocation, workforce distribution, and governance autonomy limited its overall success. Scotland’s integrated care model demonstrates potential benefits in reducing service fragmentation and improving patient-centred care; however, persistent challenges such as funding constraints, workforce shortages, and governance conflicts indicate that integration alone is not sufficient to resolve systemic healthcare inefficiencies. This study provides a perspective on Scotland’s health and social care integration, offering valuable lessons for other European countries facing similar demographic and healthcare challenges.

## 1. Introduction

During the last decade, Scotland underwent major reforms as the government and political parties agreed on the need for new laws, policies, and strategies [1]. With rising healthcare costs and an increasing number of illnesses linked to lifestyle choices, integrated care became a key policy goal [1]. The number of older population in Scotland has also increased, which requires an increase in the budget for healthcare [2,3]. A Joint Working Act was passed in 2014 to integrate Scotland’s social and health sectors [1]. This public policy Act changed how health and social care services were planned, developed, and delivered across Scotland [1]. Several studies have examined different aspects of Scotland’s health and social care integration, contributing to a deeper understanding of its policy framework, implementation challenges, and long-term impacts. Bruce & Parry (2021) provided an overview of the Public Bodies (Joint Working) (Scotland) Act 2014, detailing its legislative foundation and highlighting the structural framework of newly formed Health and Social Care Partnerships (HSCPs) [4]. The authors emphasised the Act’s goal of shifting towards a person-centred approach, ensuring individuals could live longer and healthier lives at home or in familiar environments [4]. Pearson & Watson (2018) investigated the early implementation phase of the Act, revealing structural imbalances, governance challenges, and cultural barriers that hindered its full potential. Their findings suggested that, at that stage, integration was not yet systemically embedded, relying instead on individual innovators or “boundary spanners” to drive change. They argued that despite the rhetoric of partnership working, power remained concentrated within the healthcare sector, limiting the effectiveness of the reform [5]. A more recent critical perspective from Donaldson et al. (2024) assessed whether Scotland’s integration reforms have successfully met their objectives and found that despite political and policy enthusiasm, the reforms have not fully delivered their intended outcomes, calling for even more radical changes to Scotland’s integrated care model [6]. Similarly, Hendry et al. (2021) examined key enablers and barriers to large-scale integration, emphasising the importance of relational approaches and citizen-led models [7], and highlighted that although local successes were evident, national progress appeared slower, partly due to immature data systems for measuring community-based interventions [7]. Their study advocated for greater emphasis on place-based prevention and early intervention to tackle inequalities and ensure sustainable integration [7]. Beyond Scotland, Reed et al. (2021) provided a comparative analysis of health and social care integration across the four U.K. nations, identifying common goals and policy drivers while also highlighting variations in implementation and effectiveness [8]. Their study reinforced the need for a systematic evaluation of integration outcomes, as evidence regarding its true benefits for patients and communities remains mixed [8]. While these studies offer valuable insights into Scotland’s integrated care model, our research extends this body of knowledge by applying a public policy theoretical framework, analysing the driving forces behind policy decisions, and examining how political, economic, and social factors influenced Scotland’s approach to integration.

### 1.1. Theoretical Framework

The demand for effective and efficient healthcare services has risen over the last couple of decades [9]. As resources have always been scarce, the efficiency of the healthcare system has become a significant concern for policymakers around the world [9]. While health and social care integration has been implemented in other U.K. nations, Scotland presents specific characteristics that justify a detailed analysis. The structure of the NHS (The National Health Service) in Scotland is distinct from the rest of the U.K., operating in a more centralised and publicly funded manner, which significantly influenced the design of its reforms [8]. The Scottish legislative framework, particularly the Joint Working Act of 2014, marked an approach to establishing a fully integrated governance model, distinguishing itself from England’s more market-driven and decentralised strategy [1]. The idea of integrating social and healthcare services is not new; it had been implemented in Wales and Northern Ireland way before Scotland. In Wales, major policy reforms took place, and a range of initiatives was introduced to integrate both sectors. Similarly, Northern Ireland has also been using an integrated model since 1973. Since 1999, the Scottish parliament has been devolved the challenge of healthcare administration and the integration of health and social care services has been one of the most prominent changes in Scotland ever since the National Health Services (NHS) was created [10]. Scotland had mostly followed the British health policies of the NHS, and while integration had been the theme of implementation long before, the integration of health and social services became central law in 2014, and several policies variable from the U.K. model have been implemented since 2016 [11].

However, the National Health Services (NHS) is different in Scotland compared to other countries using integrated care structures. The NHS is treated as a separate body in Scotland and is mostly public, unlike other parts of the U.K., with additional key organisational and economic differences.

### 1.2. Historical, Political, and Socioeconomic Context of Scotland

Considering the historical context, the management of NHS during the post-war period was similar to the structure in the U.K. Though the policies in Scotland were a little different, Scotland had restricted autonomy. In the early 1990s, the previous model of NHS was replaced by a new model called “internal market” that was based on market principles [12]. According to the internal market model, the country’s health boards became healthcare buyers for the hospital and the state’s citizens. By 1997, broad steps were taken by the U.K. to damage the internal market. As a result, by the year 2000, the NHS had changed its approach and adopted the method of collaboration and integration to provide better healthcare. The government that came into office in 2007 confirmed its commitment towards services that are publicly offered with a focus on mutuality involving patients, healthcare staff, and the general public as partners [13].

The political context of Scotland in 2013 and2014 reflected the decisions made by the Scottish Executive, also called the Scottish Government, who has been in office since 2007. The U.K. ministers and the secretary of state of Scotland had transferred powers to the new government, and the Scottish Parliament now had the competence of passing legislation. However, some reserved matters for which the decision-making power remained with the U.K. parliament. Among other devolved issues, the management of the health and social care sectors was now in the Scottish parliament’s hands [12].

During the time of the Joint Act public policy, which relies on the integration of the health and social care sector, the population of Scotland was 5.2 million [14]. Compared to other states of the U.K., population density has remained low in Scotland. Though the size of the population has been stable, the number of people aged 65 and above has grown gigantically in the last couple of decades. Consequently, it was considered that the healthcare sector’s expenses for the coming years would be higher than the current expenses [15].

### 1.3. Objective

This perspective paper has been constructed by aligning different characteristics of the public policy models. This examination of the reasons behind this law provides valuable insights for European countries facing similar demographic and healthcare challenges, such as Portugal, Spain, and Italy, where aging populations and fragmented social and healthcare systems present significant obstacles to integration. The authors focused this perspective paper on Europe due to the region’s shared policy landscape, governance structures, and legal frameworks, which make Scotland’s experience particularly relevant. The structural similarities in public health governance, welfare models, and legislative processes in Europe make the Scottish experience a relevant case study for potential adaptation in these contexts.

From this perspective, the authors present a critical analysis of public policy based on the integration of the social and health sectors in Scotland. The objective was to examine the underlying policy drivers, structural mechanisms, and governance implications, providing insights into the broader impact of integrated care reforms.

## 2. Methods

This study employed a qualitative research approach to analyse the integration of health and social care in Scotland through the lens of three different public policy models: public choice, institutionalism, and functionalism. The selection of those models was based on their relevance to policymaking and governance structures. These models offer distinct perspectives on how policies are formulated and implemented:

Roessler and Schmitt (2021) [16] presented that the “public choice model” of public policy is concerned with the use of economic tools to resolve traditional political science issues, like the influence of power in decision-making, roles involved in governance, and political behaviours. It is known that voters’ behaviour can influence public officials’ behaviour, and the public choice theory results reflect the social choice theory results. One of the possible reasons for deciding on integration could be the influence of public choice theory on the policymakers in Scotland [17]. By offering efficient healthcare interventions, the chances of an increasing bank of voters for the current government were high. Hence, the policymakers agreed to provide integrated services to increase the public’s trust in the new government.

The institutionalism theory presents a perspective of policymaking that suggests that individuals are embedded in institutions, and institutional support is essential for public policies [18]. Lastly, the views of functionalism emphasise the idea of offering effective healthcare to support the functional abilities of society.

### 2.1. Data Collection

The research strategy was adopted to synthesise data from multiple sources to ensure a comprehensive analysis of Scotland’s health and social care integration. Data sources included peer-reviewed journal articles retrieved from PUBMED^®^ and Google Scholar^®^ as well as Scottish government publications accessed via official government portals (www.gov.scot) between January 2012 and November 2024.

A structured search strategy was applied to retrieve relevant literature, using a combination of the following keywords: (“integration” OR “integrated care” OR “health and social care” OR “ health policy” OR “public bodies” OR “Joint Working Act” OR “integrated joint board”)AND (“policy” OR “policy analysis” OR “governance” OR “public choice”) AND (“Scotland”).

### 2.2. Data Selection and Inclusion Criteria

Data were selected based on their relevance to the study objectives. Peer-reviewed journal articles and official government documents were prioritised over grey literature. The inclusion criteria for selecting sources were: (a) Publications focused on Scotland’s health and social care integration.; (b) Studies discussing the Public Bodies (Joint Working) (Scotland) Act 2014 and its outcomes; and (c) documents analysing the impact of governance structures, funding models, and policy effectiveness.; (d) documents that incorporated qualitative or mixed methods approaches to evaluate integration. Only documents published in English were considered.

### 2.3. Data Analysis

A qualitative research approach was employed to examine Scotland’s integration efforts through the lens of public policy models. The analysis began with a comprehensive review of collected documents, ensuring familiarity with the data. Key concepts related to policy implementation, governance structures, and service integration were identified and systematically examined. These concepts were then analysed in relation to the theoretical frameworks of public choice, institutionalism, and functionalism, providing a structured understanding of how Scotland’s approach to integration aligns with or diverges from these models. Findings were synthesised to offer a contextualised interpretation of the integration process within the public policy landscape.

## 3. Results

The findings of this study were structured according to the three public policy theoretical frameworks—public choice, institutionalism, and functionalism—to provide a comprehensive understanding of Scotland’s integrated health and social care system. The results section begins by examining the influence of the public choice model, which highlights how political decision-making and economic incentives shaped the integration process. It then explores the institutionalist perspective, emphasising the role of governance structures, regulatory frameworks, and institutional arrangements in shaping the implementation and effectiveness of integrated care. Finally, the results discuss the functionalism framework, which considers how integration contributes to the overall stability and efficiency of the healthcare and social care sectors by addressing the needs of an aging population and enhancing service delivery.

Each section presents key themes identified and supported by relevant literature and policy documents.

### 3.1. Integration of Health and Social Care Under the Influence of Public Choice

The efficiency of a healthcare system is mainly analysed based on economic and social factors, whereas political influence has always been neglected [19]. However, the Joint Act of 2014 is purely a result of specific reasons examined by politicians. The government of Scotland aimed to improve the performance of the care sector by bringing greater transparency, innovation, and technology together through cooperation [13]. The main characteristic of the public choice model is that it relies on studying the behaviours in the political realm and factors that influence decision-making in politics, with individual/material self-interest guiding economic and political behaviour. The theory deduces to anticipate the way people might respond to a given situation, and the government takes decisions to match people’s choices.

The public choice model confirms that results from a political realm reflect the choice of the persons working as agents and officials with the government. As human behaviour is based on factors associated with universal explanations, the public choice model also relies on the idea that circumstances drive human behaviour. Considering the context of Scotland when the general policy was implemented, it is likely that politicians were thinking about ways to reduce health and support care expenses for the ageing population. It was realised that integrated care would help the country’s citizens stay healthy and stay in their homes for a longer time [20].

The challenge of inverse care and health inequities was widening socioeconomic barriers among the public while concentrating care provision in already high practice performance urban regions, raising concerns among rural populations [21]. The public choice of equality motivated the development of GP (General Practitioners) clusters in 2018, which could direct resources as well as staff in practice deprivation regions, thus enhancing public trust in both the quality of care and serving government [4].

The increasing cost of longer hospital stays and rising accidents and emergency burdens, in combination with declining performance, rose public concerns in 2013/14 [6]. By that time, the implementation of integrated care across the world had been beneficial in chronic management and elderly care; therefore, Scotland adopted the newly emerging approach of integrated care to address the public’s choice of quality care provision at low costs [22].

Also, efficient health and social care would satisfy residents, who would likely be willing to vote for the same government again shortly. In other words, there were several individualistic benefits associated with the decision of health and social care integration, which had made politicians of that era pass the integrated public policy. The health status largely influences the population’s attitudes towards a government.

The choice of public plays a significant role in making and breaking governments, and bringing an integrated approach forward to offer better healthcare was a favourable move. The policy implementation improved the service quality of both social and health care services as integration became a policy priority for the government.

### 3.2. Relationship of Institutionalism with the Integration of Health and Social Care

The institutionalist model emphasises the idea that the effective implementation of a public policy requires properly structured institutions. Economic instability reduces the spending power of states, and as a result, the budget allotted to the healthcare sector is limited. Due to financial constraints, delivering value-based health and support care services becomes a challenge for state governments [23]. Hence, for most European countries, providing better healthcare has become a challenge because of the limitation of funds and finances.

Conversely, institutional theory offers a conceptual framework through which the government in the office can respond to the external demands of health and social care by utilising the internal resources of organisations. The approach with which an organisation responds to external pressures largely depends on the way institutions manage the overall structure of organisations within a given sector. The way organisations are structured within a society reflects the efficiency of institutions involved in the management of organisations [24].

The creation of a public policy of integration aimed to offer high-quality healthcare so that the early prevention and intervention of disease could be made possible. The politicians of Scotland knew about the country’s socio-economic realities and were aware that many people of the country are aged above 50 years. The demographic statistics of Scotland indicated that the elderly population, over 60 years old, is increasing at a 50% rate of the overall population [25]. Furthermore, nearly 40% of the total population of Scotland is aged above 50 years. Subsequently, there was a high chance of aged people needing various care and support services to manage their lives in the next few years. Carey et al. (2014) state that countries with an ageing population must develop policies to strengthen the healthcare system [26]. In doing so, it is essential to consider different social factors related to individuals’ health and well-being. Such social factors are income level, education level, adequate housing, and access to health/wellness services. Integration could result in more vital institutions with an improved focus on delivering quality health and social services.

Strong institutions can work towards establishing efficient clinical practices, through which health outcomes for the patients can significantly improve [27]. With a focus on institutional transformation, planning and delivering healthcare/social care services could improve [24]. Considering the same assumption of strengthening institutions, policymakers of Scotland had passed the legislation of integrating health and social sectors. As per this new approach, all of the different units of the health care sector and social sector were legally required to work together to improve the lives of the population residents [28].

### 3.3. Influence of Functionalism on the Integration of Health and Social Care

The policymakers of Scotland took the theory of functionalism into account before deciding on the public policy for the healthcare sector. Planning and delivering a service are the two most essential functions in promoting superlative health and social care [29]. The theory of functionalism enables that better quality of health and social care can enhance the overall functionality of society. The critical aspects of functionalism are associated with stability, order and consensus within the community. The ideas of functionalism also consider monetary and political contributions as a mode of connecting citizens and the democratic process of the state.

The key concept of functionalism spares the sick person of societal obligations until complete well-being [30]. Scottish policy, under the influence of functionalism, spared chronically ill and elderly patients of constraints of their income and means and, while they are a social and financial dependent component of society, the Scottish government took care of their housing, social interaction, physical activities, free health, and social care as well as the provision of care at the doorstep thus fulfilling their needs and sparing them of worldly chores regardless of income and residential status through the integration of local and health authorities according to new legislations [31]. The issue of limited mobility/disability and resource deprivation has been addressed through remote consultations using telehealth [32].

Functionalism, however, refers mostly to acute sickness, which has been addressed by the Scottish integrated care system through the management of unscheduled calls and visits under NHS boards funded by separate resource allocation [33]. Chronic illness, which has been neglected by functionalism, is the centre of service delivery for the Scottish NHS. Multidisciplinary teams in both primary and secondary care, with the assistance of social care providers, address the physical, mental and social well-being of multiple morbidities within single geographical constraints, reducing the risk of long-term hospital admissions [34].

Considering the same assumption from the theory of functionalism, the integration of health and social care was aimed at improving the functionality of the NHS that is being provided in Scotland. Ever since 1997, every strategic document of the health boards of Scotland has featured integration to enhance the functionality of the healthcare system.

The idea of integration was not only focusing on the external integration of the health and social sector, but it also aimed to create internal integration within the health sector of Scotland. As integration would improve functionality for society, policymakers were willing to take steps to integrate health and social care. Conversely, the main goal of a public policy is to improve the lives of the residents of a country, and the lives can improve when the functionality of society improves. The integration of health and social sectors turned out to enhance society’s functionality and improve the health and well-being of the Scottish people [35].

### 3.4. Integration Model

The model used to integrate Scotland’s social and health care sectors is based on partnerships established across the country. These partnerships across Scotland were managed by billions of resources provided by the government body. The Joint Act of 2014 presented an integration scheme jointly prepared by the local authorities and health boards of Scotland. Within the main integration scheme, some subschemas were designed to target different regional areas of the country [36]. The government used a decentralised approach and gave both health boards and local bodies the freedom to distribute significant functions between them, which is called a lead agency arrangement. The policy also allowed health and local authorities to delegate the power of integration to a third body called the Integration Joint Board (IJB) [36].

The implementation of the Joint Act in 2014 took emergence in the form of 14 NHS boards and 32 democratically elected councils, which partnered with non-elected Integration Authorities who were responsible for the locality-based service integration of health and social care while seeking funding from NHS boards and were accountable and supported by both the boards and council [4]. Electoral and administrative clash, manifesting into funding and subsequent outcome restraints, led to the abolishment of Quality and Outcomes Framework (QOF) and development of GMS (General Medical Services) contract whereby general practitioners were controllers of locality-based GP clusters and patient data, and were responsible for patient care decisions and providing social carers access to information [37]. The ongoing challenge of health inequity and the introduction of technology into healthcare was planned to be addressed through remote care provision to rural areas through telecare in light of the Telehealth Act 2016 [38]. With the realisation of the disproportionate demand and supply of healthcare as per inverse care law, the GP contract was remodelled in 2018 to define the role of GPs, who were empowered to hire and relocate multidisciplinary teams to ensure distribution in neglected areas. General practitioners were allocated key care decisions and responsibilities for earlier consultations so as to reduce hospital admissions and emergency visits, and simultaneously, care was directed more multidisciplinary through community-based MDT (multi-disciplinary teams), including specialists, nurses, social carers, pharmacists, etc., for enhancing complex multimorbidity care quality and reducing GP and indoor workload [39]. The 2021 revision of the GP Act adopted further defragmentation of GPs by which preventive services, including vaccinations and pharmacotherapy, were returned to health boards, as well as the inclusion of MDT into primary care. Allocation of additional funds was guaranteed for the burden of services on GPs during the transition period as well as for the recruitment of sufficient MDTs [40]. The introduction of the National Care Service (NCS) 2021 led to the proposition of replacing integration authorities with community health and social care boards and centralising social care while service delivery was emphasised for elders and deprived regions [41].

#### 3.4.1. Financial Reforms

In 2016, the Central government announced the allocation of more funds to primary care. Integration joint boards could spend two-thirds of the NHS and social care budget but with restricted autonomy as they were responsible for reporting to both NHS boards and elected councils, thus limiting the effective utilisation of resources [6]. As the challenge of disproportionate need and supply emerged since 2007, the Scottish government redirected health funds in 2018 as GPs were relieved of their leadership roles and local authorities were empowered to adjust funding for higher-need populations, including elderly and chronic illnesses, and highly deprived regions, including rural and low socioeconomic regions. Funds were also allocated to cover practice expenses for clinical service delivery and the additional needs of rural practitioners [42].

#### 3.4.2. Health Reforms

Besides chronic illness and elderly care, mental health has been prioritised by Scotland as is evident by the enaction of Mental Health act 2015, mental health strategy 2017, and Mental health and well-being strategy 2022, which defined rights of mentally ill people and formulated principles for indoor and emergency psychological care provision along with strategies for effective implementation, promotion and easy access [31,43]. The challenge of the isolation and integration of community into social care for the elderly was overcome through legislations like Connected Scotland 2018 [44] and Fairer Scotland for Older People 2019. The Strategic Framework for Action on Palliative and End-of-Life Care 2015 involves the integration of families, healthcare and social care professionals for planning and delivering palliative and end-of-life care for the elderly [45].

For sustainable service delivery, the health system focused on the involvement of individuals for the management of their own health and well-being in 2016 when National clinical strategy empowered patients through educational programs and digital tools with the knowledge, skills, and confidence to self-manage their treatment plan, track progress, provide real-time feedback, and consult experts remotely thus reducing complex illness and complications related hospital admissions. Patients have autonomy over personalised care programs for lifestyle modifications, medications, and mental health well-being [46].

Scotland has long anticipated the transformation of healthcare through technology, focusing on key advancements such as remote healthcare delivery for rural communities and elderly, the empowerment of patients in tracking and managing personalised care, and the digitalisation of expert consults and triage in both primary and secondary care. Data sharing among stakeholders, including researchers and industry has been facilitated by legislation such as the National Telehealth 2012 [38], the eHealth Strategy 2017 [47], the Digital Health and Care Strategy 2018, and the revised strategies introduced in 2021 to enhance a Scotland’s position in the digital healthcare landscape [48,49].

#### 3.4.3. Public Reforms

Unlike the U.K. model of means-dependent charges of care and upper limit for free care, Scottish policy adopted the universalist approach of free health and social care for elders above 65 years in 2002 and extended this to free care for all people regardless of age, income and residential status in the Community Care Act 2018 [50]. Scottish health model acknowledged and incentivised the role of unpaid carers and directed local authorities to develop the adult carers’ support programs as well as assistance for younger carers depending on individual needs in 2013 while Carers act in 2016 required government support for Carers’ health and service integration of social carers into health amid requirement for local carer strategies [51].

Age Home and Community, a Strategy for Housing for Scotland’s Older People Act 2012–2021 tasked local governments to arrange aging-friendly affordable housing, foster community engagement, and integrate housing providers with health and social carers to provide effective elderly care at the doorstep [52]. Through Active Scotland legislation in 2018, Scottish policymakers implemented the integration of workplace and healthcare to provide a physically active environment at workplaces as well as in the outdoor environment to promote mobility in elders and reduce the healthcare burden through healthy lifestyles in the youth [53].

## 4. Discussion

This study focuses on Scotland due to its distinct approach to health and social care integration within the U.K.. Unlike other countries, which pursued a commissioning-based and competitive provider model, Scotland introduced substantial reforms with a strong public and centralised focus. The country faced unique challenges, such as a rapidly aging population and increasing care demands in rural areas, which drove innovative policy measures. The findings from the Scottish integration policies offer valuable insights into the potential benefits and challenges of integrated care systems. These results underscore the importance of coordinated efforts between the health and social care sectors to enhance care delivery while navigating systemic barriers. The evidence indicates that integrated care can significantly improve patient outcomes in several dimensions, such as shorter hospital stays and better quality of life.

Despite these positive outcomes, the inability to attract GP and MDTs to deprived areas, even with hiring autonomy, underscores the persistence of the inverse care law. This highlights the need for more innovative and radical policies to ensure the equitable distribution of healthcare resources. The limited autonomy of IJBs in managing funding was another critical issue. The proposed transition to community care and health boards, while addressing these limitations, inadvertently added financial strain due to inadequate accountability mechanisms.

Until 2019, the Scottish integration policies have successfully reduced the duration of hospital stays, but the effect on mortality remains unaltered [54]. Recent research has highlighted the psychological and behavioural benefits of integrated care, particularly in enhancing patient self-efficacy [54,55]. A comparative study by Alonso et al. [54], found that individuals receiving integrated care exhibited significantly improved self-efficacy levels, patient engagement, and adherence to treatment plans. Specifically, the study reported a 7.7% higher self-efficacy score among individuals benefiting from integrated care, suggesting that the coordinated approach to health and social services fosters greater confidence in managing personal health [54]. These findings indicate that integration enhances patient autonomy, reduces uncertainty regarding care pathways, and promotes proactive health behaviours. Additionally, integrated care has been associated with reduced psychological distress, particularly among elderly and chronically ill patients, further underscoring the holistic impact of this model on patient well-being [55,56]. Studies have also found that while integrated carers preferred safety and timely response to unscheduled care seekers, 90% of the people used NHS unscheduled care during the last year of their life and estimated that while NHS managed most of the unscheduled care burden of community, they consumed only 3.9% of unscheduled care budget emphasising cost-effective out-of-hours and end of life integrated care [57].

Literature has, however, observed the failure of GPs to relocate and recruit practitioners and MDTs in deprived areas despite complete autonomy over hiring and distribution of staff, thus requiring radical policies to combat the inverse care law [21]. Donaldson et al. observed that the limited autonomy of integrated joint boards on funding resulted in resource utilisation according to status quo directions especially in the absence of mechanisms of shifting funds and while government tried to address this issue by replacement with community care and health boards, transition process has added to the central financial burden in the absence of appropriate accountability mechanism [6]. The authors suggested that the NHS boards and IJBs could keep functioning under separate funding arrangements with accountability from representatives from health and social care as well as an elected body [6].

This study confirmed that the health and social sectors could improve both institutions and functionality within a society [58]. While integrated care has been positioned as a key strategy to enhance healthcare delivery, its effectiveness in Scotland remains a subject of debate. Although some evidence suggests that the system has demonstrated resilience despite demographic, workforce, and financial constraints, recent studies indicate that integration in its current form faces significant structural and operational barriers [7]. Donaldson et al. (2024) argue that despite policy enthusiasm, the anticipated improvements in care coordination, efficiency, and patient outcomes have not been fully realised, necessitating more radical reforms [6]. Similarly, Reed et al. (2021) highlight persistent governance challenges and funding limitations that continue to hinder progress [8]. These critiques emphasise that without stronger mechanisms for accountability, sustained investment in workforce capacity, and refined policy execution, integration alone is unlikely to meet its intended objectives.

The findings from Matos et al. further reinforce this perspective by demonstrating that, at the international level, while health and social care integration has yielded improvements in clinical outcomes, quality of life, and service quality, its impact remains inconsistent due to fragmented implementation and variable policy execution. They found that integration efforts have resulted in better access to services, reduced waiting times, and increased patient satisfaction [59]. However, they also noted that improvements in cost efficiency, collaboration, and staff perceptions often rely on limited evidence, underscoring the need for more rigorous assessment of long-term impacts. These insights align with the Scottish experience, where integration has shown potential benefits but remains constrained by financial pressures, workforce shortages, and challenges in governance structures [59].

Moreover, prevention and early intervention are important in reducing healthcare burdens and ensuring long-term sustainability. While localised and equity-driven approaches offer potential benefits, their success depends on clear financial commitments, strategic workforce planning, and the ability to adapt services to evolving population needs. To maximise integration’s impact, policymakers must address existing inefficiencies, enhance funding transparency, and reinforce decentralised decision-making processes to empower local health and social care structures.

### Strengths and Limitations of the Study

This study provides a comprehensive analysis of Scotland’s health and social care integration through the lens of public policy models, offering a unique theoretical perspective that enhances our understanding of policy drivers and outcomes. A key strength is the use of multiple frameworks—public choice, institutionalism, and functionalism—which allow for a multidimensional evaluation of policymaking and implementation. Additionally, the study synthesises evidence from a diverse range of sources, including peer-reviewed literature, government reports, and comparative analyses, ensuring a robust and well-supported discussion.

However, some limitations must be acknowledged. The study relies primarily on secondary data, which may introduce biases related to publication selection and reporting. Another limitation is that the long-term sustainability and effectiveness of Scotland’s integration model remain subject to ongoing policy and governance changes, making it difficult to draw definitive conclusions about its success. Finally, while the study applies theoretical models to explain policy choices, the complexity of real-world decision-making means that additional social, cultural, and economic factors may also have played significant roles which were not fully captured within this framework.

## 5. Conclusions

This study critically analysed the integration of health and social care in Scotland through the lenses of public choice, institutionalism, and functionalism, providing insights into the policy motivations, structural changes, and broader implications of the 2014 Joint Working Act. The findings suggest that Scotland’s approach was shaped by political and economic considerations, institutional capacity, and the functional necessity of improving healthcare access and efficiency. While integration aimed to enhance service coordination and long-term sustainability, its implementation revealed challenges related to governance, funding, and workforce distribution.

This study contributes to a deeper understanding of the decision-making processes behind integrated care and highlights both the potential and the limitations of such reforms. The Scottish experience offers valuable lessons for other European countries facing similar demographic and systemic challenges, emphasising the need for well-structured policies, sustained investment, and adaptable governance frameworks. Moving forward, continued evaluation and refinement of integrated care policies will be essential to ensure equitable and effective healthcare delivery.

## Data Availability

Requests to access the datasets should be directed to the corresponding author and will be granted upon reasonable request.
